# The Analgesic Efficacy of Nefopam in Patient-Controlled Analgesia after Laparoscopic Gynecologic Surgery: A Randomized, Double-Blind, Non-Inferiority Study

**DOI:** 10.3390/jcm10051043

**Published:** 2021-03-03

**Authors:** Soomin Lee, Seounghun Lee, Hoseop Kim, Chahyun Oh, Sooyong Park, Yeojung Kim, Boohwi Hong, Seokhwa Yoon, Chan Noh, Youngkwon Ko

**Affiliations:** 1 Department of Anesthesiology and Pain Medicine, Chungnam National University Hospital, Daejeon 35015, Korea; bimily0526@gmail.com (S.L.); hoseop75@gmail.com (H.K.); ohchahyun@gmail.com (C.O.); sycom7@gmail.com (S.P.); koho0127@gmail.com (B.H.); seohwy@cnu.ac.kr (S.Y.); 2 Department of Anesthesiology and Pain Medicine, Chungnam National University Sejong Hospital, Sejong 30099, Korea; anelee1982@gmail.com (S.L.); yeojung80@cnuh.co.kr (Y.K.); 3 Department of Anesthesiology and Pain Medicine, College of Medicine, Chungnam National University, Daejeon 35015, Korea

**Keywords:** nefopam, opioid, postoperative pain, postoperative nausea and vomiting, pain measurement, gynecologic surgical procedures, patient-controlled analgesia

## Abstract

Opioid-sparing effects of nefopam during patient-controlled analgesia (PCA) are well demonstrated. We hypothesized that postoperative pain control with an opioid-equivalent dose of nefopam as a single analgesic agent for PCA would not be inferior to fentanyl in laparoscopic gynecologic surgery. In total, 135 patients were randomly assigned to the N (nefopam 200 mg), NF (fentanyl 500 mcg + nefopam 100 mg), and F (fentanyl 1000 mcg) groups (*n* = 45 patients per group). The primary outcome was the numerical rating scale (NRS) score at rest at 6 h postoperatively, and the non-inferiority limit was set to 1. Secondary outcomes were pain severity and incidence of nausea and vomiting for 48 h postoperatively. Mean differences (MD) in primary outcome between the N and F groups were 0.4 (95% confidence interval (CI) −0.5 to 1.3). The upper limit of 95% CI exceeded the non-inferiority limit. The N group showed higher pain scores than the NF group (MD, 1.1; 95% CI, 0.3–1.9) in pairwise comparisons. No significant intergroup differences were observed in the cumulative dose of PCA usage and incidence of postoperative nausea and vomiting (PONV). In laparoscopic gynecological surgery, nefopam alone showed limited efficacy for postoperative pain control.

## 1. Introduction

Nefopam is a non-opioid, non-steroidal centrally acting analgesic and is widely to control mild to moderate pain [1]. It inhibits the reuptake of serotonin, norepinephrine, and dopamine. Intravenous nefopam rapidly produces potent inhibition of the nociceptive flexion reflex in humans [2].

Previous studies have shown that nefopam, when used in combination with opioids, reduces opioid consumption and adverse effects while providing sufficient analgesia [1,3,4]. It reduces the need for opioids by approximately 40% [5]. One study reported that, when comparing the analgesic effect of nefopam on opioids, 20 mg of nefopam exhibits equal efficacy to that of 6–12 mg of morphine [6], and another study reported that 20 mg of nefopam is equipotent to 7.5 mg of morphine [7]. The analgesic effect of nefopam alone was not lower than that of a combination of morphine and ketorolac on pain control after laparoscopic gynecological surgery [8].

Pain control after laparoscopic gynecological surgery is primarily based on patient-controlled analgesia (PCA) using opioids. Patients undergoing laparoscopic gynecological surgery show an increased incidence of postoperative nausea and vomiting (PONV) [9]. In these patients, analgesia induced with opioids further increases the probability of PONV, causing patients to stop PCA and, therefore, preventing effective pain control [10].

To the best of our knowledge, no previous study has compared the analgesic effect of PCA using nefopam alone and fentanyl alone after laparoscopic gynecological surgery. We hypothesized that analgesia with nefopam alone was non-inferior to that with fentanyl alone. Additionally, we compared to combination group made with equivalent dose of each drugs. Thus, we investigated the most effective approach to control pain without adverse effects in gynecological patients undergoing laparoscopic surgery.

## 2. Experimental Section

### 2.1. Study Design

This randomized, double-blind, non-inferiority study was conducted at the Chungnam National University Hospital, Republic of Korea, from October 2019 to June 2020. The study protocol was approved by the Chungnam National University Hospital Institutional Review Board (IRB CNUH 2019-05-032-004) and registered in the Clinical Research Information Service (KCT0004212). Written consent was obtained from participants prior to anesthesia according to the statement of the purpose and method of the study. This study was conducted in accordance with the Consolidated Standards of Reporting Trial (CONSORT) statement.

### 2.2. Participants

Study participants were patients aged 20–70 years with American Society of Anesthesiologists (ASA) physical status I or II who were scheduled to undergo laparoscopic gynecological surgery. Exclusion criteria included ASA class higher than III, refusal to participate in this study, pregnancy or plans to become pregnant, hypersensitivity or allergies to fentanyl or nefopam, or preoperative analgesic treatment.

### 2.3. Randomization and Blinding

Participants were randomly assigned to one of the three groups (N, nefopam(Acupan^®^); F, fentanyl; NF, nefopam and fentanyl) at a ratio of 1:1:1 by using a computer-generated table of random numbers with 3, 6 block sizes. To conceal the allocation, the table was uploaded through REDCap (Research Electronic Data Capture) software in our hospital and was accessible only to the researcher who prepared the study drug. The assessor who performed group assignment was blinded to the patients and the physicians who assessed outcomes. Thus, a total of 135 patients were assigned to group N (nefopam), group NF (fentanyl and nefopam), or group F (fentanyl).

### 2.4. General Anesthesia Technique and Postoperative Management

The patients received premedication with intramuscular (IM) injections of glycopyrrolate 0.2 mg/kg and midazolam 2 mg at 30 min before surgery. They also received general anesthesia with standardized monitoring. After pre-oxygenation for 3 min, general anesthesia was induced using propofol 2 mg/kg and remifentanil 1 µg/kg. After the administration of rocuronium 0.6 mg/kg, endotracheal intubation is performed when the train-of-four (TOF) count was 0. The cuff pressure of the tube was set to 20–30 mmHg, the tidal volume was adjusted to 6–8 mL/kg of the ideal body weight, and the target end tidal CO_2_ (ETCO_2_) was adjusted to 35–40 mmHg. Anesthesia was maintained with sevoflurane 1.5–2.5 vol% at 40% FiO_2_ using intravenous remifentanil (0.05–0.1 μg/kg/min) to achieve a mean arterial blood pressure and heart rate within 20% of the preinduction value.

The direct equivalent dose of fentanyl and nefopam is unknown; hence, the conversion was calculated based on the equi-analgesic intravenous dose of morphine. According to a previous study, 20 mg of nefopam provides an analgesic effect equivalent to that of 6–12 mg of morphine [6], and 10 mg of morphine provides equivalent analgesia to 100 μg of fentanyl. Thus, we set the equivalent dose as follows:

Nefopam 20 mg ≈ morphine 10 mg ≈ fentanyl 100 µg.

All PCA devices were set to administer a loading dose of 3 mL, basal dose of 1 mL/h, and bolus dose of 1 mL, with a lockout time of 15 min and a total allowable volume of 100 mL [10]. Patients in group N received nefopam 200 mg; those in group NF received fentanyl 500 µg and nefopam 100 mg; and those in group F received fentanyl 1000 µg. Each drug was mixed with 0.6 mg ramosetron in the PCA device. At the end of the surgery, an additional 0.3 mg of ramosetron apart from that included in PCA was administered intravenously to reduce PONV.

Sufficient preoperative presentations of PCA devices were performed. PCA devices were applied after obtaining informed consent from patients after sufficiently explaining to them regarding how to use and what are the side effects before surgery. In addition, after surgeries, PA nurses went up to the ward for PCA management to interview patients personally and provided education related to the use of PCA and identified the effects and the side effects of PCA once again.

The patients were observed in the post-anesthesia care unit (PACU) for an hour and then transferred to the ward, where they were continuously monitored for adverse effects, such as pain and PONV. Additional analgesics and antiemetics were administered at the discretion of the attending physician, and analgesics were generally added at the request of the patient or when the patient experienced pain with a numerical rating scale (NRS) score of 6 or higher.

The quality of postoperative functional recovery was assessed using the validated Korean version of the Quality of Recovery-40 (QoR-40) questionnaire [11]. This questionnaire consists of 40 questions that examine 5 domains of patient recovery [12]: physical comfort (12 items), emotional state (9 items), physical independence (5 items), psychological support (7 items), and pain (7 items). Each item was rated on a 5-point Likert scale as 1 (none of the time), 2 (some of the time), 3 (usually), 4 (most of the time), or 5 (all of the time). The total score ranged from 40 (very poor recovery) to 200 (outstanding recovery). An assistant researcher administered the QoR-40 two times: the day before surgery and the first day after surgery.

The brief pain inventory (BPI) was used to assess multiple dimensions of postoperative pain (Table S2).

### 2.5. Outcomes

The primary outcome was the postoperative pain score at 6 h after surgery, and the non-inferiority limit was set to 1. Secondary outcomes included pain (at rest and during coughing) and nausea severity at 1, 6, 24, and 48 h after surgery; cumulative dose of PCA usage; QoR-40 questionnaire score; brief pain inventory score; and patient satisfaction. Primary outcome was assessed by the intention-to-treat analysis, and per-protocol analysis was applied when serious deviations of the protocol were noted in questionnaire assessments at 1 h and 6 h after surgery. The pain score was assessed by the patient using the numeric rating scale (NRS; 0 = no pain, 10 = maximum pain imaginable), and nausea and vomiting were assessed by yes or no question to participants. In addition, the use of additional analgesics or the incidence of other adverse effects, such as chills, sweating, itchiness, dizziness, drowsiness, and tachycardia, was checked in the nursing record. The PCA device was collected, and the log records were stored in the research computer for evaluation of usage time, bolus frequency, and PCA discontinuation. Patient satisfaction is evaluated using a 5-point Likert scale (1 = very dissatisfied, 2 = dissatisfied, 3 = neutral, 4 = satisfied, and 5 = very satisfied).

### 2.6. Sample Size Estimation

The sample size was calculated on the basis of the primary endpoint in accordance with the non-inferiority hypothesis. In this study, an NRS pain score difference of 1 was considered as the non-inferiority margin. Based on a previous study [13], a standard deviation of 1.6 was assumed for the NRS distribution of laparoscopic gynecological surgery. To achieve a power of 80% with a risk of 0.025 for type 1 errors, the minimum number of patients required in each group was 41. Thus, a total of 135 patients were recruited to allow for dropouts. One-sided non-inferiority testing for primary outcomes was performed by comparing the 95% confidence interval of the difference between groups for the NRS score to the predetermined non-inferiority margin.

### 2.7. Statistical Analyses

The normality of continuous data was assessed using the Shapiro–Wilk test. If normality was satisfied, then independent t-tests were used for two-group comparisons (primary outcome), and one-way analyses of variance were used for three-group comparisons, with the results expressed as mean ± standard deviation. If normality was not satisfied, then Kruskal–Wallis test was performed, and the results were expressed as median (interquartile range). Post-hoc pairwise comparisons for continuous variables were performed using Tukey’s honestly significant difference test or Dunn’s Kruskal–Wallis multiple comparisons, as appropriate. Categorical data were compared using the χ^2^ test or Fisher’s exact test, as appropriate, with the results expressed as number (%). For all calculations, two-tailed *p* < 0.05 was considered statistically significant. For pairwise comparisons of categorical variables, *p*-values were adjusted using Bonferroni correction. All statistical analyses were performed using R software version 4.0.0 (R Project for Statistical Computing, Vienna, Austria).

## 3. Results

### 3.1. Study Flow Chart

**Figure 1:**
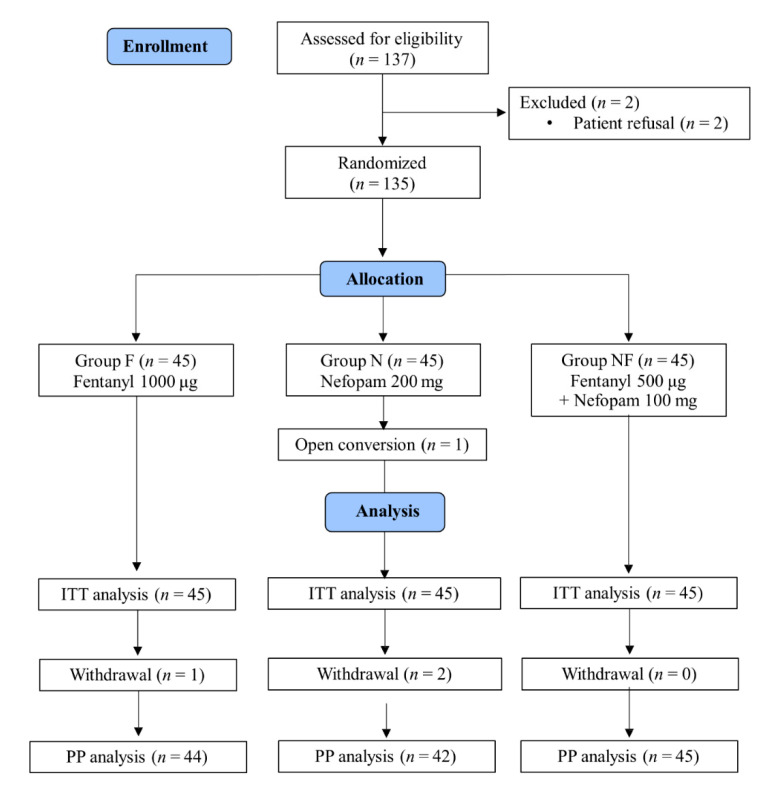
Consolidated Standards of Reporting Trials (CONSORT) flow chart. ITT analysis, intention-to-treat (ITT) analysis; PP analysis, per-protocol analysis.

A total of 137 patients were eligible for the study, two of whom refused to participate in the study. Thus, a total of 135 patients were enrolled in this study, with 45 patients randomized into each of the three groups. One patient in the N group underwent an open conversion due to a plan change during surgery, and one patient in the F group and two patients in the N group withdrew from the study before 6 h postoperatively. Thus, a total of 135 patients were included in the intention-to-treat (ITT) analysis, and 131 patients were included in the per-protocol (PP) analysis (Figure 1).

### 3.2. Demographic Data

The groups showed no significant differences in demographic and clinical characteristics (Table 1).

### 3.3. Postoperative Pain and Nausea & Vomiting

**Figure 2:**
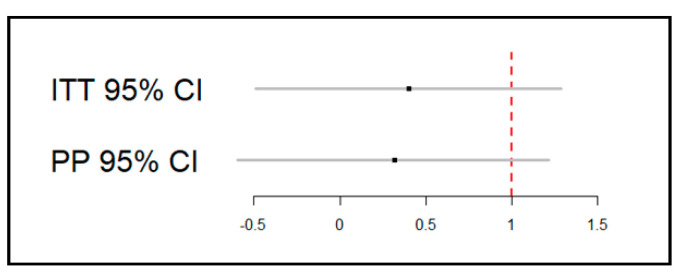
Non-inferiority margin. CI, confidence interval; ITT, intention-to-treat; PP, per-protocol.

In the ITT analysis with a non-inferiority margin of 1, the mean difference (MD) and 95% confidence interval (CI) for NRS score at 6 h postoperatively between the F and N groups was 0.4 (95% CI, −0.5 to 1.3). The MD and 95% CI for the PP analysis was 0.3 (95% CI, −0.6 to 1.2). Both analysis demonstrated that the upper limit of 95% CI exceeded the non-inferiority margin of 1 (Figure 2).

The changes in NRS scores for the three groups over time are shown in Table 2. NRS scores measured at 6 h (*p* = 0.02) and 24 h (*p* = 0.015) after surgery showed significant differences among the three groups. In pairwise comparison, the N group showed increased pain score compared with that of the NF group at 6 h (MD, 1.1; 95% CI, 0.3–1.9) and 24 h (MD, 0.9; 95% CI, 0.2–1.7) postoperatively. There was no significant difference in the dose of PCA usage over time among the three groups (Table 2), and there was no significant difference in the rates of PONV and the resulting discontinuation of PCA (Table 3).

### 3.4. PCA-Related Postoperative Complications

Except for PONV, the frequency of adverse effects, such as dizziness and drowsiness, showed significant differences among the three groups (Table 3). However, patient satisfaction, QoR-40 scores, and BPI results for each time period did not show significant differences among the three groups (Tables S1 and S2).

## 4. Discussion

Although laparoscopic surgery is less invasive than open surgery, the procedure can cause severe pain [14]. Therefore, patients who undergo laparoscopic surgery require active pain control, which is mainly achieved through PCA using opioids. However, the occurrence of PONV is more common in gynecological patients, which limits the use of opioids and complicates pain control in these patients [9]. Therefore, various methods and drugs for proper pain control in this patient population are currently being studied.

In previous studies, nefopam, a non-opioid centrally acting analgesic, at a dose of 20 mg demonstrated the same effect as that of morphine at a dose of 6–12 mg [6]. According to Kim et al., nefopam alone showed adequate postoperative analgesic effect after cardiac surgery [15]. Yoon et al. demonstrated that nefopam alone was non-inferior to morphine with ketorolac in patients undergoing gynecological surgery [8]. Fentanyl is a drug commonly used for postoperative pain control. Russo et al. compared the efficacy of fentanyl and morphine in pain control after gynecological surgery and found that, although both groups showed effective pain control, the fentanyl group patients showed faster bowel function recovery and shorter hospital stay [16]. Therefore, we compared the use of nefopam alone with fentanyl alone. Our findings suggest that pain control in PCA using nefopam alone was not non-inferior to that achieved with fentanyl in patients undergoing laparoscopic gynecological surgery. Because the fentanyl-equivalent dose of nefopam was not known and because a conversion analgesic dose of nefopam was applied, our results differ from those reported by Yoon et al. [8]. In addition, in our study, PCA was set to not exceed the daily allowance of 120 mg of nefopam. Therefore, unlike the findings of the study conducted by Kim et al. [15], which compared nefopam and fentanyl after cardiac surgery, less infusion of drugs seems to have affected the outcome.

The NRS score was high in all groups in this study. To investigate the reason for this finding, the correlation between the NRS score and drug use through PCA was examined by exploratory analysis, but no clear correlation was observed. This finding suggests that patients did not actively use PCA, despite complaining of severe pain. In addition, adverse effects and PCA usage were also compared to rule out restrictions on PCA use due to adverse effects, but no distinct difference was observed. If there were no other restrictions on the use of PCA other than adverse effects, it seems possible that the patients who reported high NRS scores did not actively press the bolus because of lower PCA compliance. In order to compensate for this point, encouragement and education promoting the use of PCA for active pain control will be more needed.

Opioids are known to induce PONV in 20–40% of patients [17,18], while nefopam is known to induce PONV in 15–30% of the patients [13]. In addition, according to the study by Oh et al. [19], the PONV incidence was 31.9% in the group using nefopam and 57.4% in the group using fentanyl for post-gynecological pain control [19], but this study did not show a significant difference among the three groups. However, the incidence of PONV in all three groups in the present study was 30–40%. Because the participants of this study constituted a high-risk group that can show a PONV incidence of up to 80% if they do not receive adequate antiemetic treatment [20], the values obtained in the present study can be considered to represent a relatively low level. It appears that the prophylactic ramosetron was effective. Moreover, previous studies have also reported that, although nefopam has an opioid-sparing effect, it may not significantly reduce PONV [4,21].

The incidence of drowsiness and dizziness is another point to be noted in this study. The rates of drowsiness and dizziness were reported to be the lowest in the N group, with significant differences from the rates in the F group for dizziness and the NF group for drowsiness. However, rather than focusing on the significance of the detailed results, it should be noted that even patients with an NRS score of 4 or higher complained of dizziness and drowsiness. In general, additional pain control is recommended for patients with an NRS pain score of 4 or higher [22], and while complaining of such strong pain, these patients also complain of the adverse effects of PCA analgesia. Therefore, a multimodal analgesic approach that combines different modalities or uses analgesics based on different mechanisms is necessary.

This study had several limitations. First, the loading and bolus dose of PCA were fixed in all patients, and better pain control would have been achieved if these doses were based on the patients’ body weight. Second, even though sufficient explanation and training were provided before and after surgery on how to use PCA, there were situations in which the patients did not use sufficient bolus doses; thus, pain control was not properly achieved. Third, because of the ethical issue, the study did not include a placebo group; thus, the true effect size of the analgesic agents were not acquirable within current study. Moreover, the exact equivalent dose of nefopam to fentanyl was not known; hence, the conversion analgesic dose of nefopam was applied. Thus, additional studies on the equivalent and safe dose with larger samples are required.

## 5. Conclusions

In laparoscopic gynecological surgery, nefopam alone was not non-inferior to fentanyl alone as an analgesic agent for PCA for postoperative pain control. Additionally, PCA alone showed limited efficacy for postoperative analgesia in our study. Future studies combining additional analgesics as a multimodal approach for PCA are required.

## Figures and Tables

**Table 1:** Demographic and clinical characteristics.

	Group F	Group N	Group NF
	(*n* = 45)	(*n* = 45)	(*n* = 45)
Age (yr)	46.0 (41.0; 49.0)	46.0 (41.0; 50.0)	43.0 (30.0; 48.0)
ASA status (1/2)	22/23	24/21	21/24
Height (cm)	159.3 ± 5.4	157.7 ± 6.3	159.7 ± 6.3
Weight (kg)	57.0 ± 8.2	56.5 ± 6.1	59.4 ± 7.9
BMI (kg/m^2^)	22.4 ± 2.7	22.8 ± 2.8	23.3 ± 2.9
Smoking	1 (2.2%)	0 (0.0%)	2 (4.4%)
Previous PONV	0 (0.0%)	2 (4.5%)	0 (0.0%)
Surgery type			
TLH	27 (60.0%)	24 (54.5%)	19 (42.2%)
Ovarian cystectomy	12 (26.7%)	12 (27.3%)	18 (40.0%)
Salpingo-oophorectomy	5 (11.1%)	7 (15.9%)	6 (13.3%)
Others	1 (2.2%)	2 (4.4%)	2 (4.4%)
Port type			
One port	34 (75.6%)	29 (65.9%)	35 (77.8%)
Two ports	11 (24.4%)	13 (29.5%)	10 (22.2%)
Three ports	0 (0.0%)	2 (4.5%)	0 (0.0%)
Open	0 (0.0%)	1 (2.2%)	0 (0.0%)
Duration of anesthesia (min)	94.0 (80.0; 105.0)	100.0 (82.0; 111.0)	85.0 (75.0; 101.0)
Remifentanil dose (µg/kg/min)	0.1 (0.1; 0.1)	0.1 (0.1; 0.1)	0.1 (0.1; 0.1)

ASA, American Society of Anesthesiologists; BMI, Body Mass Index; PONV, Postoperative Nausea and Vomiting; TLH, Total Laparoscopic Hysterectomy.

**Table 2:** Change in numerical rating scale (NSR) scores in the three groups over time and the dose of patient-controlled analgesia.

	Group F	Group N	Group NF	Overall-*p*	Group F vs. N	Group F vs. NF	Group N vs. NF
	(*n* = 45)	(*n* = 45)	(*n* = 45)		*p* *	*p* *	*p* *
NRS on resting							
PACU	7.0 (6.0; 8.0)	8.0 (6.0; 10.0)	6.0 (5.0; 8.0)	0.063			
6 h	5.0 (4.0; 8.0]	6.0 (5.0; 7.0)	5.0 (3.0; 6.0)	0.020	0.627	0.213	0.027
24 h	4.0 (2.0; 5.0)	4.0 (4.0; 6.0)	3.0 (2.0; 5.0)	0.015	0.051	0.521	0.017
48 h	3.0 (2.0; 4.0)	3.0 (2.0; 4.5)	3.0 (2.0; 4.0)	0.328			
NRS on coughing							
PACU	5 (11.1%)	3 (6.7%)	3 (6.7%)	0.789			
6 h	8.0 (6.0; 9.0)	8.0 (6.0; 10.0)	8.0 (6.0; 9.0)	0.297			
24 h	6.0 (5.0; 8.0)	7.0 (5.0; 8.0)	6.0 (4.0; 8.0)	0.120			
48 h	5.0 (3.0; 6.0)	6.0 (4.0; 7.0)	4.0 (3.0; 6.0)	0.092			
Cumulative doseof PCA (mL)							
PACU	6.0 (5.0; 6.0)	6.0 (6.0; 6.0)	6.0 (5.0; 6.0)	0.057			
6 h	16.0 (13.0; 19.0)	15.0 (13.0; 18.0)	15.0 (13.0; 18.0)	0.742			
24 h	40.0 (33.0; 49.5)	37.5 (34.0; 45.0)	38.0 (32.0; 45.0)	0.859			
48 h	72.0 (62.0; 85.0)	67.0 (60.0; 77.0)	70.0 (56.0; 79.0)	0.292			

* *p*-values < 0.05 were considered significant. PCA, patient-controlled analgesia; PACU, post-anesthesia care unit; PONV, postoperative nausea and vomiting.

**Table 3:** Postoperative complication and patient-controlled analgesia withdrawal due to postoperative nausea and vomiting.

	Group F	Group N	Group NF	Overall-*p*	Group F vs. N	Group F vs. NF	Group N vs. NF
	(*n* = 45)	(*n* = 45)	(*n* = 45)		*p* *	*p* *	*p* *
Total complications	32 (71.1%)	26 (57.8%)	34 (75.6%)	0.17			
PONV	18 (40.0%)	14 (31.1%)	16 (35.6%)	0.678			
PCA withdrawal d/t PONV	6 (13.3%)	3 (6.7%)	6 (13.3%)	0.509			
Other complications	31 (68.9%)	18 (40.0%)	32 (71.1%)	0.004	0.011	1	0.006
Dizziness	25 (55.6%)	11 (24.4%)	20 (44.4%)	0.01	0.005	0.399	0.076
Hypotension	1 (2.2%)	1 (2.2%)	1 (2.2%)	1			
Pruritus	3 (6.7%)	2 (4.4%)	0 (0.0%)	0.37			
Sweating	5 (11.1%)	3 (6.7%)	3 (6.7%)	0.789			
Drowsiness	19 (42.2%)	8 (17.8%)	23 (51.1%)	0.003	0.021	0.526	0.002
Tachycardia	1 (2.2%)	0 (0.0%)	0 (0.0%)	1			
Urticaria	1 (2.2%)	1 (2.2%)	0 (0.0%)	1			

* *p*-values < 0.017 were considered significant. PONV, postoperative nausea and vomiting.

## Data Availability

The data presented in this study are available on request from the corresponding author.

## Supplementary Materials

The following are available online at https://www.mdpi.com/2077-0383/10/5/1043/s1, Table S1: Patient satisfaction score and QoR-40 scores, Table S2: Brief pain inventory short for (BPI-SF) scores.
