# Supplementary material for: The Analgesic Efficacy of Nefopam in Patient-Controlled Analgesia after Laparoscopic Gynecologic Surgery: A Randomized, Double-Blind, Non-Inferiority Study
Source: J Clin Med. 2021 Mar 3;10(5):1043. doi: 10.3390/jcm10051043 (PMC7959469; doi:10.3390/jcm10051043)
Supplement: Supplementary file 1 [file jcm-10-01043-s001.pdf]

Table S1. Patient satisfaction score and QoR-40 scores

	Group F (*n* = 45)	Group N (*n* = 45)	Group NF (*n* = 45)	*p*
Satisfaction score				
● PACU	3.0 [2.0; 4.0]	3.0 [2.0; 3.0]	3.0 [2.0; 4.0]	0.211
● 6 h	3.0 [3.0; 4.0]	3.0 [3.0; 4.0]	3.0 [3.0; 4.0]	0.465
● 24 h	4.0 [4.0; 4.0]	4.0 [3.0; 4.0]	4.0 [3.0; 4.0]	0.519
● 48 h	4.0 [4.0; 5.0]	4.0 [4.0; 4.0]	4.0 [3.0; 4.0]	0.668
QoR-40 domain				
● Comfort	57.0 [55.0;59.0]	56.0 [52.0;58.0]	55.0 [54.0;59.0]	0.302
● Emotions	42.0 [39.0;43.0]	39.0 [35.0;44.0]	38.0 [36.0;42.0]	0.187
● Physical independence	25.0 [24.0;25.0]	25.0 [21.0;25.0]	24.0 [21.0;25.0]	0.209
● Patient support	34.0 [32.0;35.0]	32.0 [29.0;35.0]	31.0 [27.0;35.0]	0.122
● Pain	34.0 [33.0;35.0]	34.0 [32.0;35.0]	33.0 [31.0;35.0]	0.46
Global QoR-40 scores	188.0 [181.0;195.0]	184.0 [171.0;195.0]	181.0 [172.0;189.0]	0.088

Table S2. Brief pain inventory short for (BPI-SF) scores

group	F (*n* = 45)	N (*n* = 45)	NF (*n* = 45)	*p*
bpi_1	29 (64.4%)	32 (71.1%)	28 (62.2%)	0.651
bpi_2	37 (84.1%)	40 (88.9%)	42 (93.3%)	0.356
bpi_3	8.0 [6.0; 9.0]	8.0 [6.0; 9.0]	8.0 [6.0; 8.0]	0.749
bpi_4	2.0 [2.0; 3.0]	3.0 [2.0; 4.0]	2.0 [2.0; 3.0]	0.201
bpi_5	4.6 ± 1.7	5.1 ± 1.6	4.3 ± 1.6	0.068
bpi_6	3.7 ± 1.7	3.7 ± 2.1	3.4 ± 1.6	0.697
bpi_7_0	14 (31.1%)	15 (33.3%)	13 (28.9%)	0.902
bpi_7_1	6 (13.3%)	10 (22.2%)	8 (17.8%)	0.544
bpi_7_2	0 (0.0%)	1 (2.2%)	1 (2.2%)	1
bpi_8	70.0 [50.0;80.0]	70.0 [50.0;80.0]	70.0 [50.0;80.0]	0.976
bpi_9_1	8.0 [5.0; 8.0]	7.0 [5.0; 9.0]	7.0 [5.0; 8.0]	0.914
bpi_9_2	5.0 [3.0; 8.0]	5.0 [4.0; 7.0]	5.0 [2.0; 8.0]	0.948
bpi_9_3	6.0 [4.0; 8.0]	7.0 [5.0; 8.0]	7.0 [3.0; 8.0]	0.196
bpi_9_4	7.0 [5.0; 8.0]	7.0 [5.0; 9.0]	7.0 [3.0; 9.0]	0.889
bpi_9_5	3.0 [1.0; 6.0]	5.0 [2.0; 7.0]	3.0 [1.0; 6.0]	0.282
bpi_9_6	5.0 [3.0; 7.0]	6.0 [3.0; 8.0]	4.0 [2.0; 7.0]	0.445
bpi_9_7	6.0 [3.0; 8.0]	6.0 [3.0; 8.0]	5.0 [3.0; 8.0]	0.623
